# Preprocedural Mean Platelet Volume Level Is a Predictor of In-Stent Restenosis of the Superficial Femoral Artery Stents in Follow-Up

**DOI:** 10.1155/2018/4572629

**Published:** 2018-04-01

**Authors:** Kurtulus Karauzum, Ulas Bildirici, Emir Dervis, Irem Karauzum, Canan Baydemir

**Affiliations:** ^1^Department of Cardiology, Kocaeli University Medical Faculty, Kocaeli, Turkey; ^2^Department of Biostatistical, Kocaeli University Medical Faculty, Kocaeli, Turkey

## Abstract

**Background:**

The mean platelet volume (MPV), the most commonly used measure of the platelet size, is a cheap and easy-to-use marker of the platelet activation. We aimed to evaluate the relationship between preprocedural MPV and other hematologic blood count parameters and in-stent restenosis in patients with superficial femoral artery (SFA) stenting.

**Methods and Results:**

The consecutive 118 patients who successfully underwent endovascular stenting of the SFA were enrolled retrospectively in the study. The mean follow-up was 23 ± 12 months. The in-stent restenosis was observed in 42 patients (35.6%). There were no statistically significant differences between the restenosis group and no-restenosis group in terms of age, gender, and smoking (*p*=0.116, *p*=0.924, and *p*=0.428, resp.). In the restenosis group, the MPV level was markedly higher than that in the no-restenosis group, and it was statistically significant (*p* < 0.001). According to the ROC curve analysis, the optimal cutoff value of the MPV to determine the restenosis was >8.7 fL, and the level of the MPV >8.7 fL was a strong predictor of the restenosis (*p* < 0.001) in logistic regression analysis.

**Conclusions:**

The measurement of the preprocedural MPV levels may help to identify high-risk patients for development of the in-stent restenosis. These patients may benefit from an aggresive antiplatelet therapy and close follow-up.

## 1. Introduction

Endovascular treatment of the superficial femoral artery (SFA) has been successfully performed as an alternative to surgery for a long time [1]. It has been demonstrated that use of the stents in the SFA has a high procedural success and less complications [2]. Although there have been advances in the peripheral stent technology in recent times, the most important issue of the stenting is still in-stent restenosis in long-term follow-up, and its treatment is difficult [3]. Therefore, prediction and prevention of the in-stent restenosis of the SFA are very important for disease management. The platelets are known to play a very important role in the pathogenesis of the cardiovascular diseases [4]. The platelets are heterogeneous in size and activity [4]. Larger platelets are denser and contain more alfa granules which can release prothrombotic substances, including platelet factor 4, P-selectin, and platelet-derived growth factor (PDGF), a chemotactic and mitogenic factor contributing to vascular neointimal proliferation [5, 6]. Overall, all of these mechanisms may play a crucial role in the peripheral in-stent restenosis process by accelerated neointimal proliferation. The mean platelet volume (MPV), the most commonly used measure of the platelet size, is a cheap and easy-to-use marker of the platelet activation [7, 8]. In previous studies, the MPV has been shown to be elevated in many cardiovascular diseases, and increased the MPV levels in atrial fibrillation (AF), acute myocardial infarction (AMI), and cerebrovascular infarction have been associated with increased platelet activity [9–12]. In this study, we aimed to evaluate the relationship between preprocedural MPV and other hematologic blood count parameters and in-stent restenosis in patients who had undergone successful endovascular treatment with stenting of the SFA.

## 2. Methods

### 2.1. Patients

The patients who successfully underwent endovascular stenting of the SFA between March 2013 and May 2016 in Cardiology Department of Kocaeli University Hospital which is a high-volume specialized PAD (peripheral artery disease) clinic were enrolled retrospectively in the study. The study group consisted of 118 consecutive patients who discharged from the hospital with optimal medical therapy. Exclusion criteria were the use of warfarin or other anticoagulants for any reason, acute coronary syndromes in the last 6 months, atrial fibrillation, overt/active hematological, immunological, renal, and hepatobiliary disease, active infection, malignant diseases, and end-stage other diseases with life expectancy less than one year. The Cardiology Clinic of Kocaeli University Hospital has a detailed clinical database of the patients with PAD. The collected data included demographic information and medical history such as age, gender, previous cerebrovascular events, coronary artery disease (CAD), hyperlipidemia, chronic renal dysfunction, hypertension, and diabetes mellitus. Baseline biochemical and hematological analysis including haemoglobin, haematocrit, MPV, platelet count, red cell distribution width (RDW), and platelet distribution width (PDW) levels were recorded in all patients. The estimated glomerular filtration rate (eGFR) was calculated using the modification of diet in renal disease (MDRD) formula. Concomitant medical therapy including statin and cilostazol was computed as positive if the patients had these medications. The leg symptoms of the patients were assessed according to the Fontaine claudication classification. Before stenting, the characteristics of the SFA lesions were classified according to the Trans-Atlantic Inter-Society Consensus II (TASC II).

The study protocol was approved by the local institutional investigation committees.

### 2.2. Medical and Interventional Treatment Protocols

All patients were taking a loading dose of 325 mg aspirin before the procedure and 75 mg clopidogrel daily for 7 days before the procedure or 300 mg for 3 days before the procedure or 600 mg within 24 hours before. Then, all patients took clopidogrel (75 mg per day) at least one month after discharge and were on chronic aspirin therapy (100 mg per day). Arterial access was gained through an ipsilateral or contralateral common femoral artery puncture. Contralateral puncture was applied in patients with ostial SFA occlusion. Intentional subintimal access was achieved using either a hydrophilic 0.035 inch wire or a 0.018 inch guidewire. Intravenous heparin (100 units/kg) was administered at the start of all the procedures. Primary angioplasty was performed with appropriately sized noncompliant balloons with inflation times ranging from 60 to 180 seconds after the SFA recanalization. We implanted a nitinol stent in accordance with the American College of Cardiology/American Heart Association guidelines to patients who had a residual pressure gradient >10 mm·Hg, residual stenosis >30%, and/or flow-limiting dissection after balloon therapy. The stents were self-expandable and had the diameter approximately 2 mm larger than the reference diameter. Selective stenting with self-expanding bare-metal nitinol stents (the Protégé EverFlex Self-Expanding Peripheral Stent System, Plymouth, MN, USA) was performed. The overlapping segment was approximately 5–10 mm in patients treated with a double stent. Stent implantation was never performed in the middle or distal third of the popliteal artery.

### 2.3. Follow-Up and Definitions

In our clinic, after revascularization, the routine controls of the patients with SFA stents are evaluated at 1, 3, 6, and 12 months and then every 6 months with duplex scan and peripheral angiography in patients who have recurrent leg symptoms. Restenosis was defined as >2.4 of the peak systolic velocity index by duplex at the target lesion and/or >50% grade of obstruction in the SFA stent by peripheral angiography.

### 2.4. Laboratory Measurements

The whole blood samples for the measurements of the MPV and other hematologic parameters were collected in EDTA-containing tubes. These blood samples were drawn in the morning after an overnight fasting during hospitalization before the peripheral intervention. All samples were processed within 30 minutes after blood collection from the same machine. Both intra-assay and interassay coefficients of variation for all measurements were <5%.

### 2.5. Statistical Analysis

All statistical analyses were performed using IBM SPSS for Windows® version 20.0 (SPSS, Chicago, IL, USA). Kolmogorov–Smirnov tests were used to test the normality of data distribution. Continuous variables were expressed as mean ± standard deviation or median (25th–75th percentiles), and categorical variables were expressed as counts (percentages). Comparisons of normally distributed continuous variables between the groups were performed using Student's *t*-test, and comparisons of nonnormally distributed continuous variables between the groups were performed using the Mann–Whitney *U* test. Comparisons of categorical variables between the groups were performed using Fisher's exact chi-square test, Yates' chi-square test, and Monte Carlo's chi-square test. Receiver operating characteristic (ROC) curve analysis was performed to determine sensitivity and specificity of different cutoff points for the MPV and PDW to predict in-stent restenosis. The most appropriate cutoff point was chosen according to ROC analysis, and the area under the curve (AUC) was calculated. Logistic regression analysis of the MPV and PDW for predicting in-stent restenosis was performed. A two-sided *p* value < 0.05 was considered statistically significant.

## 3. Results

**Figure 1:**
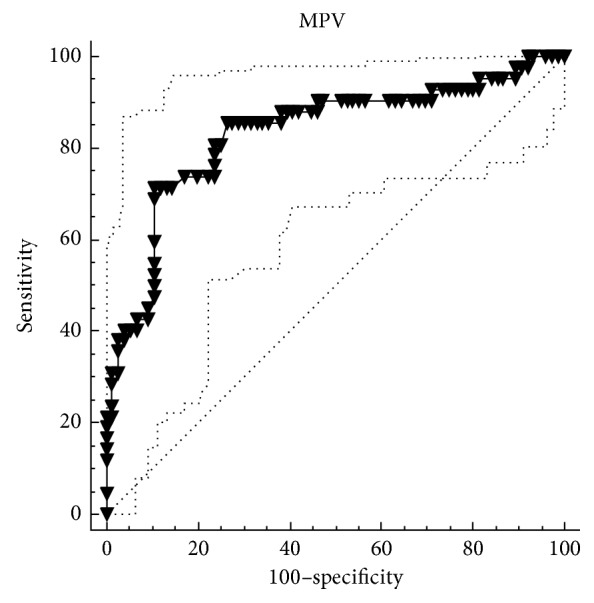
Optimized cutoff value was determined for the MPV using the ROC curve analysis.

**Figure 2:**
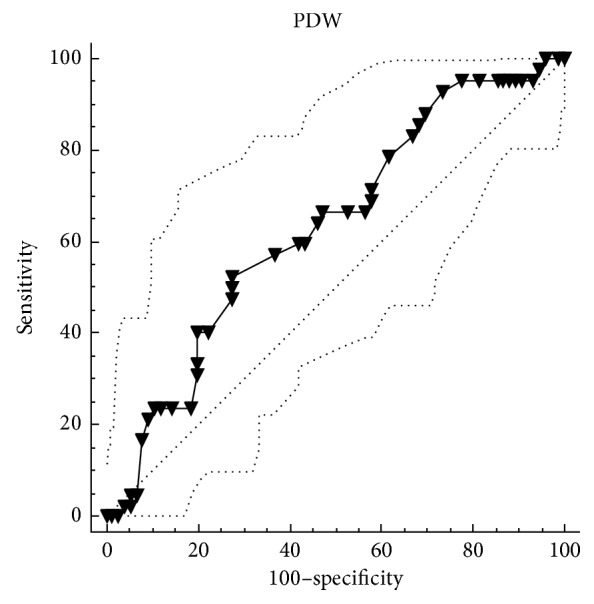
Optimized cutoff value was determined for the PDW using the ROC curve analysis.

A total of 118 patients (82.2% male) were included in the study. The mean age of the patients was 63.5 ± 10 years. The mean follow-up was 23 ± 12 months. The in-stent restenosis of the SFA was observed in 42 patients (35.6%) in the study population. There were no statistically significant differences between the restenosis group and no-restenosis group in terms of age, gender, and smoking (*p*=0.116, *p*=0.924, and *p*=0.428, resp.). Additionally, there were no differences between these two groups in terms of comorbidities such as hypertension, diabetes mellitus, hyperlipidemia, and heart failure. In the restenosis group, mean eGFR was 62.5 ± 23.1 ml/min, and it was 54.8 ± 24.5 ml/min in the no-restenosis group, but it was not statistically significant (*p*=0.259). Moreover, there were no differences between the two groups in terms of concomitant medical therapy including statin and cilostazol (*p*=0.915 and *p*=0.527, resp.). Baseline characteristics and clinical features of the patients in the restenosis group and no-restenosis group are shown in Table 1. The mean haemoglobin level was 12.9 ± 1.8 mg/dl in the restenosis group and 12.4 ± 2.1 mg/dl in the no-restenosis group, and it was not statistically significant (*p*=0.346). The mean platelet count was 235 (185–272) mm/10^3^ in the restenosis group and 220 (190–309) mm/10^3^ in the no-restenosis group, and it was not statistically significant (*p*=0.571). The PDW level was higher in the restenosis group than that in the no-restenosis group (18.55 fL (17.40–19.32) versus 17.80 fL (16.90–18.70)), and it was statistically significant (*p*=0.018). In addition, in the restenosis group, the MPV level was markedly higher than that in the no-restenosis group, and it was statistically very significant (*p* < 0.001). All of the hematological blood count indices in study patients with and without restenosis are shown in Table 2. The ROC curve analysis of the MPV and PDW levels is shown in Figures 1 and 2. According to the ROC curve analysis, the optimal cutoff value of the MPV to determine the restenosis was >8.7 fL and the optimal cutoff value of the PDW was >18.8 fL. The AUC of the MPV was 0.832 (*p* < 0.001, 95% CI 0.752–0.895), and the AUC of the PDW was 0.632 (*p*=0.013, 95% CI 0.538–0.719). However, the level of the MPV >8.7 fL was a strong predictor of the restenosis (*p* < 0.001) in logistic regression analysis, but the level of PDW >18.8 fL was not statistically significant (*p*=0.210). The logistic regression analysis of the MPV and PDW for predicting in-stent restenosis is shown in Table 3.

## 4. Discussion

Endovascular treatment with stenting is an acceptable alternative to bypass surgery in patients with SFA lesions [13]. In-stent restenosis is a common and well-known complication of these procedures [14]. Unfortunately, it is a difficult complication, and its management is challenging for physicians [14]. These patients often suffer recurrent extremity-threatening ischemia and severe claudication at the time of in-stent restenosis of the SFA [15]. Therefore, it is very important to know the clinical features that may cause in-stent restenosis. It has been demonstrated that many parameters including diabetes mellitus, smoking, dyslipidemia, long complex lesions, and age are predictors of the in-stent restenosis after SFA interventions in the follow-up [16, 17]. The rates of in-stent restenosis can be decreased by taking several precautions to these clinical features. Although these precautions are taken, in-stent restenosis of the SFA is observed to have high rates especially in mid- and long-term follow-up [18–20]. Therefore, knowing the additional predictors of the in-stent restenosis of the SFA which can be used easily and cost-effectively in daily practice may improve the clinical outcomes such as stent patency. In this context, we studied the relationship between basic preprocedural hematologic blood count parameters and in-stent restenosis of the SFA in our study. We found that there is a relationship between increased the MPV levels and in-stent restenosis of the SFA. A hematological blood count parameter which is named “MPV” reflects the platelet size and activity [4]. Larger platelets are metabolically and enzymatically more active than smaller platelets, containing more prothrombotic materials, with increased thromboxane and glycoprotein IIb/IIIa receptor expression. They show greater aggregability and decreased inhibition of aggregation by prostacyclin [8, 21, 22]. All of these ways are important mediators of the atherosclerosis, inflammation, thrombosis, and neointimal proliferation. Some of these substances such as platelet factor 4, P-selectin, glycoprotein IIb/IIIa, and PDGF are technically challenging and expensive and cannot be used in daily practice [23]. But the MPV is a cheap, easy-to-use marker and straightforward. In many previous studies, it has been shown that elevated MPV is associated with atherosclerotic diseases including AMI, unstable angina, and cerebrovascular events [9–12]. In our analysis, we showed that the MPV levels were markedly higher in patients with SFA stent restenosis than those without restenosis (Table 2). In the ROC curve analysis, the optimal cutoff value of the MPV to determine the restenosis was found to be >8.7 fL. Then, logistic regression analysis showed that the MPV level of >8.7 fL was a strong predictor of the SFA stent restenosis with a very good discriminative ability (AUC: 0.832, *p* < 0.001). In addition, there was not statistically significant relationship between in-stent restenosis and other hematological parameters such as haemoglobin, platelet count, and RDW (Table 2). Although PDW levels of the restenosis group were significantly high with the Mann–Whitney *U* analysis, the PDW level of >18.8 fL was not statistically significant in the logistic regression analysis (Table 3). Yang et al. demonstrated that the MPV was a predictor of restenosis after percutaneous transluminal coronary angioplasty (PTCA) in patients with stable and unstable angina pectoris within 6 months [24]. The MPV was significantly elevated in the restenosis group, compared with that in the no-restenosis group in the study of Yang et al. (8.75 ± 0.99 fL versus 8.04 ± 0.74 fL, *p* < 0.001) [24]. Additionally, there were an inverse correlation platelet count and restenosis. But, the remaining hematological parameters were not significantly different in the two groups as well as in our study [24]. Norgaz et al. found the relationship between the preprocedural MPV and coronary in-stent restenosis in 6 months [25]. The MPV was higher in the in-stent restenosis group than that in the no-restenosis group (8.28 ± 0.71 fL versus 7.63 ± 0.74 fL, *p*=0.001) [25]. An MPV value of ≥8.4 fL was associated with in-stent restenosis in this study [25]. Another study demonstrated that there was a positive correlation between the MPV and coronary artery restenosis after PTCA (*p*=0.01) [26]. Similarly, there was no association between restenosis and haemoglobin, haematocrit, and platelet count [26]. In contrast to all of these data, Koza et al. interestingly showed that higher MPV levels significantly reduced lower limb in-stent restenosis by approximately 34% (*p*=0.045) [7]. They could not explain this result that demonstrated a favorable effect of higher MPV on primary stent patency [7]. This result is contradictory, and it cannot contribute accurate data to the literature. We found that a simple and cheap parameter of hematologic blood count, MPV, is associated with primary SFA stent patency. In-stent restenosis was observed to have higher rates in patients with increased MPV levels. Dual antiplatelet therapy may be considered to be given to these patients for longer time. Thus, our study will contribute to the literature with its results.

## 5. Limitations

There are some limitations of our study. As this study is a single-center retrospective analysis, our results are dependent on the accuracy of the recorded hospital data with a small sample size. The MPV levels may be affected by diabetes mellitus, CAD, or hypertension, but there is no statistically significant difference between the groups in terms of these comorbidities. The MPV measurements can be affected by the time of blood collection and the type of anticoagulants in the blood tubes during transportation. However, in our study, all blood samples were collected in the morning before the procedure and analyzed within 30 minutes; therefore, the possibility of such an effect is minimal. Also, as our study is a retrospective analysis, prospective, multicenter, randomized-controlled studies are required to validate the reliability of these results.

## 6. Conclusion

In previous studies, it has been demonstrated that the MPV, a parameter of platelet size and activity, is a predictor of the restenosis after coronary PTCA and stenting as well as many cardiovascular diseases [4, 24–26]. To our knowledge, this is the first time we found that there is a relationship between elevated MPV levels and in-stent restenosis of the SFA stents in our study. High preprocedural MPV levels were associated with in-stent restenosis in follow-up. The MPV is cost-effective and easily available in all modern hospital laboratories, and the results can be learned in very short time. Thus, the measurements of the preprocedural MPV levels may help to identify high-risk patients for the development of the in-stent restenosis after successful SFA stenting intervention. Overall, these patients may benefit from an aggressive antiplatelet therapy after intervention and intensified controls of other risk factors, and they should be followed closely.

## Figures and Tables

**Table 1:** Baseline clinical characteristics of the study patients with and without in-stent restenosis.

Characteristic	With restenosis (*n*=42)	Without restenosis (*n*=76)	*p* value
Age (years)	61.5 ± 7.6	64.1 ± 9.4	0.116
Male gender, *n* (%)	35 (83.3%)	62 (81.6%)	0.924
Smoking, *n* (%)	34 (81.0%)	67 (88.2%)	0.428
Comorbidities, *n* (%)			
Hypertension	33 (78.6%)	57 (75.0%)	0.833
Diabetes mellitus	23 (54.8%)	33 (43.4%)	0.323
Hyperlipidemia	17 (40.4%)	33 (43.4%)	0.899
Coronary artery disease	25 (59.5%)	42 (55.3%)	0.800
Heart failure	6 (14.3%)	8 (10.5%)	0.759
Previous cerebrovascular event	2 (4.8%)	2 (2.6%)	0.615
eGFR (mL/min/1.73 m^2^)	62.5 ± 23.1	54.8 ± 24.5	0.259
Concomitant medical therapy, *n* (%)			
Statin	27 (64.3%)	57 (67.1%)	0.915
Cilostazol	21 (50.0%)	32 (42.1%)	0.527
Fontaine claudication classification, *n* (%)			0.156
Stage I	0 (0%)	0 (0%)	
Stage II	17 (40.5%)	40 (52.6%)	
Stage III	17 (40.5%)	30 (39.5%)	
Stage IV	8 (19.0%)	6 (7.9%)	
TASC lesion classification, *n* (%)			0.355
Type A	8 (19.0%)	23 (30.3%)	
Type B	14 (33.3%)	27 (35.5%)	
Type C	17 (40.5%)	24 (31.6%)	
Type D	3 (7.1%)	2 (2.6%)	

eGFR = estimated glomerular filtration rate; TASC = Trans-Atlantic Inter-Society Consensus.

**Table 2:** Hematological features in study patients with and without in-stent restenosis.

Parameter	With restenosis (*n*=42)	Without restenosis (*n*=76)	*p* value
Haemoglobin (mg/dl)	12.9 ± 1.8	12.4 ± 2.1	0.346
RDW (%), median (IQR)	15.55 (14.90–16.73)	15.70 (14.63–16.98)	0.798
Platelet count (mm/10^3^), median (IQR)	235 (185–272)	220 (190–309)	0.571
PDW (fL), median (IQR)	18.55 (17.40–19.32)	17.80 (16.90–18.70)	0.018
MPV (fL), median (IQR)	9.10 (8.39–10.65)	7.60 (6.98–8.33)	<0.001

IQR = interquartile range; MPV = mean platelet volume; PDW = platelet distribution width; RDW = red cell distribution width.

**Table 3:** Logistic regression analysis of the MPV and PDW for predicting in-stent restenosis.

Variable	*β*-coefficient	Odds ratio (95% CI)	*p* value
PDW >18.8 fL	0.731	2.078 (0.662–6.522)	0.210
MPV >8.7 fL	3.597	36.503 (9.635–138.285)	<0.001

MPV = mean platelet volume; PDW = platelet distribution width.
